# Analysis of the Factors Influencing the Intention to Share Information: Word-of-Mouth About Fast-Food Restaurants

**DOI:** 10.3390/foods13223559

**Published:** 2024-11-07

**Authors:** Gabriel Usiña-Báscones, Andrés García-Umaña, Iván Veas-González, Doris Celi-Pinza, Mary Llamo-Burga, Ignacio López-Pastén, Oscar Ortiz-Regalado, Nelson Carrión-Bósquez

**Affiliations:** 1 Facultad de Ciencias Sociales, Educación Comercial y Derecho, Universidad Estatal de Milagro, Milagro 091050, Ecuador; gusinab@unemi.edu.ec; 2 Facultad de Administración y Economía, Escuela de Diseño e Innovación Tecnológica, Universidad de Tarapacá, Arica 1000000, Chile; andresgarcia.dis@gmail.com; 3 Departamento de Administración, Facultad de Economía y Administración, Universidad Católica del Norte, Antofagasta 1270709, Chile; nelson.carrion@ucn.cl; 4 Carrera Administración de Empresas, Escuela de Ciencias Administrativas y Contables, Sede Santo Domingo, Pontificia Universidad Católica del Ecuador, Quito 170143, Ecuador; dceli917@pucesd.edu.ec; 5 Escuela Profesional de Ingeniería en Agronegocios, Universidad Nacional de Cajamarca, Cajamarca 06001, Peru; mllamob@unc.edu.pe (M.L.-B.); oortizr@unc.edu.pe (O.O.-R.); 6 Facultad de Economía y Negocios, Universidad Santo Tomás, Antofagasta 1240000, Chile; ilopez7@santotomas.cl

**Keywords:** fast food, physical ambiance, hedonic value, utilitarian value, consumer satisfaction, WOM

## Abstract

In a highly competitive market, word-of-mouth (WOM) has become one of the most effective ways to attract new customers, as consumer opinions are seen as reliable and have a direct impact on their consumption habits. Based on this premise, the present study aimed to analyze the factors that influence fast-food restaurant consumers in Chile in their intention to share word-of-mouth information. A quantitative approach was adopted for this analysis, using a cross-sectional and correlational design, which included 739 Chilean fast-food consumers, who were given a 25-item questionnaire. This questionnaire was developed from previous research in the field and validated by a panel of experts in marketing and research. The data analysis was conducted using the statistical software Smart PLS 4, allowing for the evaluation of the model’s convergent and discriminant validity, as well as facilitating hypothesis testing through structural equation modeling. The results showed that the physical atmosphere of the restaurants generates both hedonic and utilitarian value for consumers, which increases their satisfaction and reinforces their intention to recommend these restaurants. In conclusion, this study provides valuable insights into consumer behavior, offering a solid foundation for strategic decision making that could enhance the positioning of restaurants in the market and create a loyalty cycle among customers.

## 1. Introduction

The intention to share information in the context of fast-food restaurants, also known as word-of-mouth (WOM), refers to the consumers’ predisposition to recommend an establishment following a consumption experience [1,2]. This phenomenon can be influenced by several factors, such as Physical Ambiance (PA) [3], Hedonic Value (HV) [4], and Utilitarian Value (UV) [5], which can lead to a satisfying consumption experience [3]. According to Whelan et al. [6], WOM has become a key metric in marketing to measure Consumer Satisfaction (CS). In this sense, the likelihood that a user will recommend a fast-food restaurant depends on the perceived experience, which, in turn, impacts the establishment’s visibility and reputation in the market [7].

In relation to the aforementioned variables, PA encompasses both the tangible and intangible elements of the establishment, such as lighting, design, color, sounds, and scents, which create a sensory impression on the consumer [3]. On the other hand, HV refers to the pleasure and enjoyment that the customer experiences through these elements [4]. Meanwhile, UV is associated with functionality and the fulfillment of practical expectations, related to location, speed of service, and the value-for-money ratio [8], factors that have become influential elements in CS. Therefore, CS refers to the positive or negative evaluation of the experience that a consumer has acquired [9].

In light of the aforementioned, investigating how these variables interact and influence the intention to share word-of-mouth information has become an urgent need for both academia and fast-food businesses. Identifying the factors that shape consumer behavior is essential for strategic decision making and the development of competitive advantages [10].

The fast-food industry has become a significant global phenomenon [9,11]. In Latin America, fast-food consumption generates approximately 64.5 million dollars, with projected growth of 4.5% by 2028 [12]. This trend is also reflected in Chilean commerce, where the fast-food industry has seen a 12.8% increase in the second quarter of 2024 [13]. These figures highlight the growing relevance of the fast-food industry in Chile, underscoring the need for comprehensive research into Chilean consumer behaviors. In this context, competition between establishments is constant, and the WOM effect exerts significant influence on consumer decision making [14], a phenomenon some authors have described as “the best zero-cost advertising” [15,16].

The relationships between the factors that generate WOM have been the subject of numerous studies, with much of the scientific community agreeing on the importance of contributing results to this field of study, as positive WOM has limited impact, but negative WOM can be detrimental [10,17,18]. Despite growing evidence on the influence of PA, HV, UV, and CS on WOM, there is a lack of studies exploring how these factors specifically interact within the fast-food restaurant context [2,4,7,11].

Given the growing importance of the fast-food industry within the global economy, the objective of this study is to explore the factors influencing the intention to share information about fast-food restaurants. To achieve this objective, this study aims to address the following research questions: (a) How does PA influence CS, HV, and UV in consumers? (b) How do HV and UV influence CS? (c) How do HV and UV influence WOM? And (d) How does CS influence WOM?

This article is structured into six key sections: the first introduces the topic and sets out the main objectives of this study; the second provides a comprehensive review of the relevant literature, contextualizing this work in previous research; the third describes the methodology employed, detailing the approach and techniques used to collect and analyze the data; the fourth presents the results obtained; the fifth offers a discussion of the findings in relation to previous studies; and the sixth concludes, summarizing the main findings of this study, implications, limitations, and recommendations for future lines of research.

## 2. Literature Review

### 2.1. Intention to Share Information (Word-of-Mouth—WOM)

It begins with the concept of word-of-mouth (WOM), which can be defined as informal communication between people about a product, brand, or service [1,2]. WOM specifically refers to the likelihood that a user will share their consumption experience with others, whether positively or negatively [19]. In other words, the intention to recommend refers to the probability or willingness of consumers to suggest an establishment to other potential customers [5]. In this context, WOM is a key indicator of post-consumption behavior and is frequently used as a metric of customer loyalty and satisfaction [20,21,22].

The literature review shows that WOM in the context of fast-food consumption is influenced by various factors, including the following: (a) product characteristics, (b) consumption experiences, and (c) the establishment’s PA [6]. Regarding product or service characteristics, novelty, complexity, and exclusivity can increase consumer interest in generating WOM [23,24,25,26]. On the other hand, consumption experiences have a significant impact on customer satisfaction and the intention to recommend the product or service to their close social circle [2,27,28,29]. Meanwhile, PA enhances the establishment’s reputation and fosters consumer trust [30,31], thus stimulating HVs and UVs among users [32].

WOM not only reflects a positive evaluation of the consumption experience but also the desire to support and promote a restaurant and its activity [33]. Therefore, positive WOM enhances the reputation of an establishment [24,34,35,36]. In light of this finding, identifying the factors that lead consumers to generate WOM has become an urgent necessity that must be addressed by the scientific community [32,37]. In this regard, studies such as that conducted by Ali and Javed [38] have emphasized the need for future research to identify potential mediating factors that influence WOM. Meanwhile, other authors highlight the importance of conducting studies that determine the relationship between CS and the intention to share word-of-mouth information [29,39].

### 2.2. Physical Ambiance

Physical Ambiance (PA) refers to the internal and external design of an establishment, comprising tangible elements such as lighting, spatial arrangement, aromas, and other environmental factors [40], which can create a unique experience for the consumer and, in turn, affect the satisfaction an individual derives from a product or service [5,41,42]. Therefore, when the PA of an establishment is well designed, it can enhance CS [43,44,45].

The literature suggests that PA can generate emotions that significantly impact CS [46,47], providing unique experiences, impressions, and sensations that create deep and memorable connections with consumers [32,48]. However, studies such as those conducted by Faizan et al. [49] and Jee-Hoon [50] contradict this idea, asserting that there is no relationship between the two variables, clarifying that CS is influenced by other factors. Additionally, Kamel and Mansour [51] encourage further studies that explore the relationship between PA and CS. Therefore, this study aims to test the following hypothesis:

**H1.**  *The PA of fast-food restaurants influences CS.*

On the other hand, the perception of sophistication and PA can generate a positive attitude towards an establishment, fostering a pleasant and entertaining experience for the consumer [32,52,53]. Therefore, any added value generated through physical elements in an establishment positively stimulates consumers’ sensory experiences [54]. These experiences are defined in the academic and scientific literature as “hedonic value”. In this regard, studies such as those conducted by Lee et al. [55] and Rodhiah et al. [56] suggest that PA is related to the HV of the establishment, which can increase behavioral intentions toward fast-food consumption. However, research conducted by Güzel and Yonca [57] contradicts this statement, arguing that there is no relationship between PA and HV due to the presence of other factors. Given the theoretical contradictions identified in the existing literature, this study aims to test the following hypothesis:

**H1a.**  *The PA of fast-food restaurants influences the HV of consumers.*

Additionally, other studies indicate that consumers value the fact that the money spent on the dining experience is related to the quality of the product and service received [58]. This experience, generated in the consumer, is defined as “utilitarian value”. Studies such as those conducted by Izquierdo et al. [59] and Lee et al. [55] confirm that elements of UV, such as location, speed, quality, and price, are related to the PA of an establishment. However, Nieminen [60] states the opposite, determining that UV does not influence CS and affirming that there is no relationship between PA and UV. Nevertheless, Tariyal et al. [33] recommend investigating the factors associated with the aforementioned variables, which is why the present study aims to test the following hypothesis:

**H1b.**  *The PA of fast-food restaurants influences the UV of consumers.*

### 2.3. Hedonic Value

HV represents the emotional and experiential component a consumer perceives when acquiring or using a product or service, extending beyond its functional utility or tangible benefit [4]. This concept is particularly important in the fields of marketing and behavioral economics, where the goal is to understand not only the rational motivations for purchasing but also the emotions that consumers associate with the product, brand, or consumption process itself [7]. Thus, HV helps elucidate how sensory and affective aspects can influence purchasing decisions and the emotional connection with a brand or product, giving the company a differential advantage in the market [34]. By fostering a deeper emotional relationship with the consumer, HV becomes a fundamental element for increasing CS and brand loyalty, which is especially relevant in competitive markets where emotional factors can serve as a critical differentiator [61,62,63].

The literature suggests that HV has a direct impact on consumers’ perception of satisfaction, as it appeals to the creation of unique and unforgettable experiences [52]. In the study conducted by Nohekhan and Barzegar [34], it is shown that HV not only raises CS by meeting emotional needs but also enhances sensory experiences derived from interacting with the product or service, thus generating a more complete and gratifying consumption experience [54]. Additional research argues that high HV can set an establishment apart from the competition by providing consumers with an experience that is perceived as unique and personalized, increasing their likelihood of repeat visits or recommending it to others [32,64]. These hedonic experiences not only capture the customer’s attention but can also heighten perceived value, which, in turn, translates into higher levels of CS and loyalty. However, the role of HV in customer satisfaction is not free from debate as some researchers claim that HV does not influence CS because factors such as functional quality, price, and convenience may have a greater weight in the perception of satisfaction [65]. This contrast in empirical findings highlights an ambiguity in the literature on the influence of HV on CS, underlining the need for further exploration of this relationship. To this end, the present study proposes the following hypothesis:

**H2.**  *The HV influences fast-food CS.*

The relationship between HV and WOM intentions has been explored in several studies, suggesting that consumers may be more inclined to recommend products that evoke feelings of pleasure and enjoyment [10]. This tendency is rooted in the idea that positive emotional experiences enhance the overall perception of a product, creating a desire to share it with others as a form of social endorsement or validation [41]. Pleasurable and memorable aspects of a product can serve as key motivators for WOM, as consumers often seek to share experiences that have resonated with them on a personal level [66]. However, the degree to which HV influences WOM may vary depending on the nature of the hedonic experience, with different types of pleasure (sensory vs. emotional satisfaction) potentially impacting the conditions under which consumers are inclined to engage in WOM behavior [67].

Despite the general consensus on the positive effect of HVs on WOM, there is some academic debate regarding this relationship [68,69]. For example, Hartatin and Simanjuntak [70] present a contrasting view, suggesting that HV does not necessarily influence WOM intentions. This divergence highlights the complexity of the HV-WOM relationship, indicating that other factors, such as individual personality traits, consumer social context, and specific product features, may also play a crucial role in determining WOM behavior. Furthermore, while some studies propose that a high perceived HV may trigger WOM due to the uniqueness and memorability of the experience [68], Hartatin and Simanjuntak’s [70] findings emphasize the need for a more nuanced exploration of the variables mediating this relationship. In order to address the controversies presented in the literature, this study aims to test the following hypothesis:

**H2a.**  *HV influences the WOM of fast-food consumers.*

### 2.4. Utilitarian Value

UV is a key concept in consumer behavior, centered on the functional benefits a product or service offers to meet specific, practical needs of the consumer [16]. UV is often associated with attributes such as efficiency, functionality, and reliability, as it emphasizes the product’s role in problem solving and task completion [8]. This type of value is crucial in categories where the consumer prioritizes utility over emotional or experiential factors [7]. Thus, UV highlights essential factors such as time and cost savings, convenience, and comfort, which can significantly impact the consumer’s decision making process, especially in highly functional product segments [16,71]. By focusing on the tangible benefits, UV provides consumers with a sense of control and satisfaction from knowing they are making practical, economically sound choices [72].

The importance of UV in CS has been widely discussed in the recent literature, with various authors exploring its role in enhancing the overall consumption experience. For instance, studies conducted by Li and Yang [44] and Nusrat and Huang [45] suggest that, when a product’s performance surpasses consumer expectations, it leads to a heightened sense of satisfaction. This finding implies that UV directly impacts CS by fulfilling or even exceeding the functional promises made to the consumer [16]. Additionally, Teo et al. [7] argue that UV has a particularly strong effect on satisfaction in functional product categories, where the primary motivation for purchase is the product’s ability to fulfill specific needs. In these contexts, the assurance of reliability and effectiveness becomes a critical determinant of consumer loyalty and repeat purchases, underscoring the role of UV in fostering long-term satisfaction and brand preference [61,73,74].

Despite the general agreement on UV positive influence on CS, some authors present an alternative view. Abu et al. [75] contend that the elements constituting UV may not significantly impact CS, suggesting that, in certain cases, functional attributes alone may not be enough to drive satisfaction. Due to the discrepancies evidenced in the literature, this study seeks to test the following hypothesis:

**H3.**  *UV influences fast-food CS.*

Moreover, UV has been shown to positively impact consumers’ trust intentions within WOM contexts. When a product or service effectively meets basic functional needs, consumers are more inclined to share their experiences with others, as UV fosters a sense of reliability and satisfaction that they may want to communicate. This idea implies that UV not only fulfills practical expectations but also strengthens consumer confidence in recommending the product or service. In other words, the more a product aligns with utilitarian expectations, the more likely it is to generate positive WOM, as consumers view it as a trustworthy and valuable choice worth sharing [10,33]. This connection underscores the importance of UV as a driver for organic promotion and brand advocacy within communities.

The effect of UV on WOM can be understood as a natural extension of the satisfaction derived from the functional benefits of a product or service [16]. When consumers perceive that these benefits fulfill their expectations, they may feel compelled to recommend the product to others, which enhances the WOM effect [14]. In this regard, WOM is not merely a spontaneous outcome but a calculated response to UV, as consumers consciously decide to share positive experiences based on the product’s practical value [72]. Nevertheless, the literature addressing the relationship between UV and WOM remains limited, and more research is needed to explore how functional satisfaction translates into WOM behaviors across various product categories and consumer demographics [76]. This gap suggests an opportunity to better understand how and when UV-driven satisfaction becomes a catalyst for WOM.

Given this lack of comprehensive studies on the UV-WOM relationship, several researchers have called for further exploration in this area. Somba et al. [14], Baber et al. [15], and Catubig et al. [16] emphasize the need for future research to investigate this connection, particularly in contexts where UV plays a significant role in consumer decisions. To fill this gap in the literature, the following hypothesis is proposed:

**H3a.**  *UV influences the WOM of fast-food consumers.*

### 2.5. Consumer Satisfaction

Consumer Satisfaction (CS) refers to the experience a consumer has after purchasing and using a product or service [77]. This dynamic process is influenced by the medium- and long-term interactions and perceptions that a consumer experiences [78]. CS is a central concept in marketing that drives customer relationship management, as it influences consumer loyalty [79]. According to Ahmed et al. [9], the level of satisfaction a consumer experiences is a key factor that conditions the likelihood of recommending a product or service to their close social circle.

Given the aforementioned points, CS is a fundamental element related to WOM behavior, facilitating plausible growth for establishments [2,19]. Therefore, when consumers are satisfied with a product or service, they are likely to share positive experiences with friends, family, and acquaintances. These types of recommendations are highly valuable for businesses, as WOM is perceived as a reliable source of information by other consumers and can amplify a brand’s reach without incurring additional advertising costs [38].

The literature review affirms that WOM is an inherent consequence of a product or service that stimulates CS; however, the importance lies in whether the WOM effect is positive or negative [30,77,80]. Although several scholars have proven the relationship between these two variables, Fitriyah and Hussein [80] argue that there is a lack of studies proving otherwise. In this regard, Sakiyama et al. [17] and Zhong and Zhong [18] call for future research to explore the relationship between CS and WOM, thereby contributing theoretically to this field of knowledge. Based on the points raised, this study proposes the following hypothesis:

**H4.**  *CS influences the WOM of fast-food consumers.*

### 2.6. Research Model

**Figure 1:**
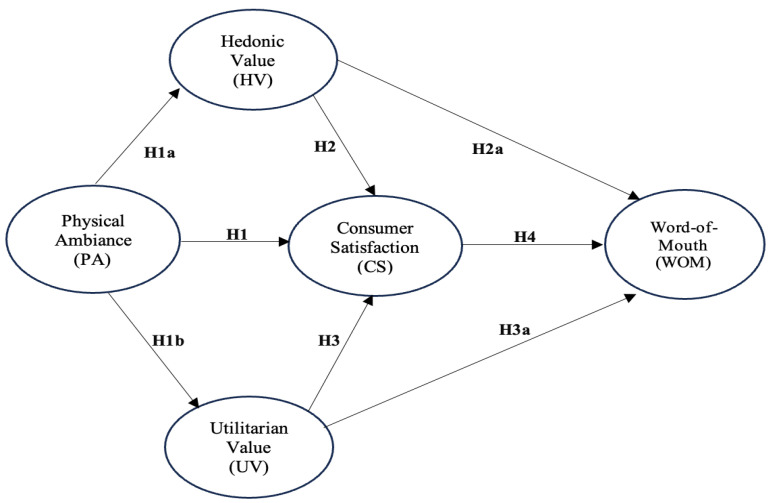
Research Model.

The research model to be hypothesized in the present study is described below (Figure 1).

## 3. Method

### 3.1. Instrument Design and Data Collection

In order to empirically verify the hypotheses formulated in the research model, a quantitative study of correlational scope and cross-sectional design was carried out, through a questionnaire consisting of 25 questions and adapted to Spanish. This questionnaire was applied to fast food consumers in Chile. Prior to the data collection, the survey was validated by two marketing experts and two research experts, none of whom raised objections. After obtaining the approval of the specialists, a pilot test was carried out with 30 participants to assess the level of understanding of the instrument.

The study sample consisted of 739 Chilean consumers. In order to obtain a sample that was both accessible and economically viable, participants were selected through a non-probabilistic convenience sample. The study population consisted of customers of the branches of the three most prominent fast-food chains in the country. Given the considerable size and popularity of these chains, as well as their franchise business model, it was hypothesized that the data collected from these locations would be representative of other similar branches and other fast-food restaurants in Chile. The respondents were required to be at least 18 years of age and were approached in person at the exits of the restaurants. The questionnaire was delivered through a QR code, and only customers who had eaten inside the restaurants were included in this study. The evaluation and validity of the structural model were analyzed using Smart PLS 4 statistical software [81].

### 3.2. Measures

The items for each of the variables were adapted based on measurement scales used in previous studies. A five-point Likert scale was used (1 representing “strongly disagree” and 5 “strongly agree”). Four questions were adapted to measure the PA variable [82], five questions were adapted for HV, and four for UV [83]. For the CS variable, three questions were adapted, and three were also adapted for WOM [83]. Review Appendix A Table A1.

### 3.3. Statistical Procedure

For data analysis, the Partial Least Squares—Structural Equation Modeling (PLS-SEM) approach was used [84], which allows for the estimation and evaluation of the proposed theoretical model. The data evaluation was conducted following the procedures and critical values established by Hair et al. [85]. Unlike covariance-based SEM (COV-SEM), PLS-SEM requires information about residual distributions, measurement scales, and sample sizes [85]. Smart PLS-SEM is considered appropriate for analyzing complex research models that serve as an estimation framework, incorporating related theories and empirical data. Following Leguina [86]’s suggestion, a two-step approach was adopted. In the first step, the measurement model was tested through internal consistency, convergent validity, and discriminant validity, and in the second step, the structural model was evaluated for hypothesis testing using SEM.

## 4. Results

### 4.1. Demographic Characteristics of the Participants

Regarding the demographic characteristics of the sample, Table 1 shows that, out of a total of 739 participants, 47.5% were men and 52.5% were women. The average age within the sample was 24 years, with a range between 18 and 41. The respondents were primarily single, accounting for 47.2%, while 25.7% were married. In terms of educational level, the majority of respondents held a university degree (54%), 41.67% had completed secondary education, and 1.48% had only primary education.

In terms of monthly income (in Chilean pesos), 49.6% indicated that they were employed, while 50.4% were not. A total of 67.11% reported monthly incomes between CLP 0 and CLP 200,000, 25.6% between CLP 200,000 and CLP 300,000, and 1.48% over CLP 601,000. Regarding spending on fast-food restaurants, 19.64% reported spending up to CLP 30,000, 62.11% up to CLP 40,000, and 11.8% spending CLP 50,000 or more (See Table 1).

### 4.2. Estimation of the Measurement Model

To test the reliability and convergent validity of the measurement model, statistical tests such as Cronbach’s Alpha, Composite Reliability (CR), and Average Variance Extracted (AVE) were used. As shown in Table 2, the values obtained for Cronbach’s Alpha and CR exceeded 0.70, meeting the threshold established in the literature [85]. Likewise, each of the standardized factor loadings of the variable indicators is greater than 0.70 [87,88,89], providing additional evidence of the satisfactory reliability of the study variables. Finally, convergent validity was ensured by assessing whether the AVE values were greater than 0.5. According to Hair et al. [85], this value is the minimum acceptable level to consider adequate convergent validity. Therefore, the results show good levels of internal consistency of the variables and adequate convergent validity. Recent research has determined that when AVE values are greater than 0.50 and lower than CR values, convergent validity is further confirmed [90]. See Table 2.

To test discriminant validity, the criterion proposed by Fornell and Larcker [90] was used, which states that the square root of the AVE should exceed the correlation values for each pair of constructs in the hypothesized model. As shown in Table 3, all the square roots on the main diagonal exceed the correlation values of the other constructs in the model. To evaluate discriminant validity, the criteria proposed by Fornell and Larcker and the HTMT ratio [90] were applied. According to Fornell and Larcker’s approach, the square root of the AVE for each variable must be greater than its correlations with other variables. Table 3 shows that all square roots (displayed in bold on the main diagonal) exceed the correlations with their corresponding constructs in the model. Regarding the HTMT ratio, the literature recommends a maximum acceptable value of 0.90. As observed in Table 3, all HTMT values are below 0.90.

### 4.3. Structural Equation Modeling (SEM): Model Fit and Hypothesis Testing

After analyzing the psychometric properties of the instrument, the structural model was estimated. The SmartPLS software [81] was used, with 5000 bootstrapped samples to evaluate the causal relationships and their significance levels. Additionally, the software was used to obtain results related to the variance of the dependent variables attributed to the explanatory variable and other variables of interest [91].

In the evaluation of the predictive capacity of the structural model, the R² values were initially assessed following the guidelines of Falk and Miller [92]. According to their indications, R² values should be greater than 0.1 to be considered acceptable; lower values, even if statistically significant, do not meet the standards. Moreover, the Standardized Root Mean Square Residual (SRMR) was calculated. Henseler et al. [93] note that SRMR is a goodness-of-fit measure for PLS-SEM that helps avoid model misspecifications. A value below 0.08 is considered indicative of a good fit [85].

**Figure 2:**
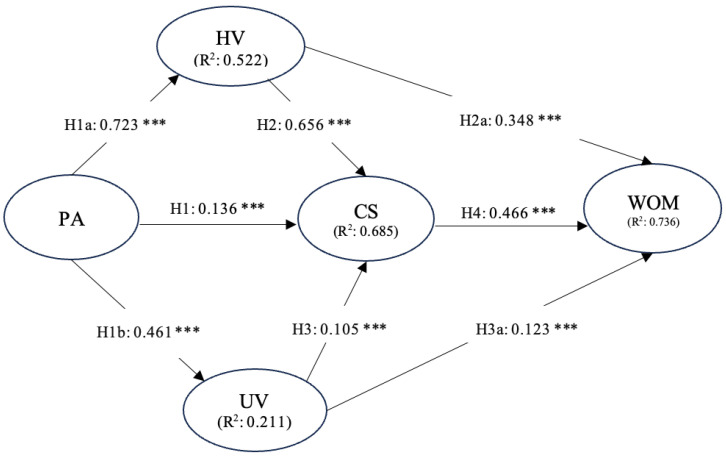
Hypothesized model. (*p* < 0.05; ***).

Furthermore, the results obtained in the relationships among the five variables in the hypothesized model allowed for the acceptance of all the proposed hypotheses in this study. The estimated values obtained through PLS-SEM show that PA (β = 0.136, *p* < 0.001) influences CS, HV (β = 0.723, *p* < 0.001), and UV (β = 0.461, *p* < 0.001). Additionally, the results indicated that HV (β = 0.656, *p* < 0.001) and UV (β = 0.105, *p* < 0.001) influence CS. Likewise, the HV variable (β = 0.348, *p* < 0.001) and UV (β = 0.123, *p* < 0.001) influence WOM. Finally, the analysis showed that CS also influences WOM (β = 0.466, *p* < 0.001) (See Table 4 and Figure 2).

## 5. Discussion

To address the research questions posed in this study, the discussion of the findings is presented in four sections: (a) How does physical ambiance influence consumer satisfaction, hedonic value, and utilitarian value in consumers? (b) How do hedonic and utilitarian values influence consumer satisfaction? (c) How do hedonic and utilitarian values influence WOM? And (d) How does consumer satisfaction influence WOM?

### 5.1. Influence of Physical Ambiance on Consumer Satisfaction, Hedonic Value, and Utilitarian Value

This study revealed that both the interior and exterior design of an establishment contribute to creating positive experiences for consumers. Based on this finding, H1 is accepted, meaning that the PA of fast-food restaurants influences CS. This result demonstrates that PA generates emotions that significantly impact CS [46,47], as a well-designed establishment enhances CS [43,44,45]. This finding aligns with studies that found a positive relationship between these two variables [46,47] and contradicts studies such as Faizan et al. [49] and Jee-Hoon [50], which found no relationship between the two variables.

Additionally, this study revealed that intangible elements, such as music, lighting, and aromas characteristic of a fast-food restaurant, are important to consumers. Therefore, H1a is accepted, meaning that the PA of fast-food restaurants influences the HV of consumers. This finding indicates that the arrangement of tangible and intangible elements in a fast-food restaurant enhances consumers’ sensory experiences and differentiates the establishment from the common market. This finding supports the conclusions of Picot-Coupey et al. [64] and Nittala and Moturu [65] and opposes Hülya and Sevilay [94], who found no relationship between these two variables.

Moreover, this study confirmed that PA in a fast-food restaurant is related to UV, meaning that consumers value elements such as location, service speed, and price. As a result, H1b is confirmed, meaning that the PA of fast-food restaurants influences the UV of consumers. This finding corroborates that consumers value a dining experience associated with UV [8]. This finding supports the conclusions of Lee et al. [55] and refutes the position of Nieminen [60], who stated that there is no relationship between UV and CS.

### 5.2. Influence of Hedonic and Utilitarian Value on Consumer Satisfaction

The study results showed that HV and UV influence CS, thereby accepting hypotheses H2 and H3. This finding confirms that HV and UV influences fast-food CS, highlighting that sensory experience, functionality, efficiency, cost, and the practical utility of a product or service influence CS [8]. This finding supports studies that indicate high HV and UV influence fast-food CS [7,32,64,65] and contradicts Abu et al. [75], who stated that UV and HV do not influence consumption satisfaction.

### 5.3. Influence of Hedonic and Utilitarian Value on WOM

This study demonstrated that sensory satisfaction, combined with key factors like time and money savings, comfort, and convenience, are crucial elements in ensuring the intention to share word-of-mouth information. Therefore, hypotheses H2a and H3a are accepted, confirming that HV and UV influence WOM in fast-food consumers. This finding shows that experiences that meet consumers’ specific needs and generate positive emotions are the most effective means of word-of-mouth advertising, where fast-food consumers share these experiences with their close social circle [8,66]. These findings align with those of Osei et al. [61] and Rominingtyas [4], who determined that HV and UV promote the WOM effect [55,59], and contradict Hartatin and Simanjuntak [70], who stated that there is no relationship between HV and WOM. Additionally, this study addresses the recommendations of Somba et al. [14], Baber et al. [15], and Catubig et al. [16], who highlighted the need for future research to expand the literature on the relationship between UV and WOM.

### 5.4. Influence of Consumer Satisfaction on WOM

The findings demonstrated that CS influences the intention to share word-of-mouth information. Therefore, H4 is accepted, meaning that CS influences the WOM of fast-food consumers. This finding demonstrates that the ambiance of an establishment stimulates consumers’ sensory and emotional experiences, thereby increasing CS. This finding aligns with the conclusions of Sakiyama et al. [17] and Zhong and Zhong [18], who determined that WOM is viewed as a reliable source of information by other consumers and can increase a brand’s reach without additional advertising costs [9,38]. Additionally, this hypothesis contributes to addressing the literature gap in this field of study, as noted in recent research [77,88].

## 6. Conclusions

This study explored the factors that influence the intention to share information about fast-food restaurants in Chile, known as “Word of Mouth” (WOM), a key behavior for promoting a business without direct advertising costs. Among the elements evaluated, PA and HV and UV are fundamental for generating satisfactory experiences that motivate consumers to recommend the restaurant.

The research demonstrated that PA, understood as the arrangement of tangible and intangible elements such as design, lighting, music, and aromas, significantly impacts CS, fostering a positive and memorable experience. Additionally, this study highlights the importance of HV, linked to the pleasure and sensory enjoyment experienced by the consumer, and UV, associated with functionality and service efficiency, such as speed or value-for-money ratio. Both values directly influence CS, a key determinant for WOM.

This analysis reveals that satisfied consumers, in addition to experiencing an emotional connection with the environment, are more likely to share their positive experiences, strengthening the restaurant’s reputation within their social circles. Thus, WOM becomes a powerful tool that restaurants can use to improve their positioning, creating a loyalty cycle that benefits both the consumer and the establishment.

### 6.1. Practical, Theoretical, and Social Implications

From a practical perspective, this study provides valuable recommendations for fast-food restaurant managers and owners in managing the customer experience. PA elements, such as music, design, and aromas, not only have a direct impact on CS but also stimulate the HV and UV of consumers. This action enhances CS and promotes WOM intention. This finding suggests that investing in the establishment’s ambiance can lead to increased WOM recommendations. Restaurants should focus not only on food quality but also on creating an atmosphere that generates positive emotions and differentiates them from competitors. Additionally, ensuring service efficiency and offering a proper value-for-money ratio strengthens utilitarian value, thereby increasing customer satisfaction and loyalty.

From a theoretical perspective, this study expands the understanding of factors that influence WOM intentions by proposing a more comprehensive framework that connects PA with HV and UV, which, in turn, are linked to CS and the willingness to recommend. By addressing a gap in the existing literature, particularly within the Chilean context, this study emphasizes the need for further research on how these factors interrelate across different cultures and market types. Moreover, this work offers a new perspective on the role of HV, suggesting that sensory experience can be as significant as functionality in fast-food consumption. This finding contributes to a more holistic view of the consumer experience in this sector, proposing that sensory and emotional stimuli are as fundamental to generating satisfaction and loyalty as functional aspects.

From a social perspective, the results emphasize the role of consumers as active agents in shaping a restaurant’s reputation through WOM. In an environment where consumption decisions are increasingly influenced by opinions shared on social media and within close circles, this study suggests that establishments should focus on generating comprehensive positive experiences for their customers, both functionally and emotionally. This approach can promote responsible consumption behavior, fostering a recommendation culture based on quality and experience rather than just convenience.

### 6.2. Limitations and Future Research

The present study was based on a sample of fast-food restaurant consumers in Chile, which limits the generalizability of the findings to other cultural contexts or types of food establishments. Furthermore, a cross-sectional design was used, which captured consumer behavior at a specific point in time, without considering how these factors might evolve over time. Other limitations of this study were the economic status and the age of the participants, as most were around 24 years old. This age restriction hinders the ability to generalize the results to the entire population of fast-food consumers in Chile, which includes a broader and more diverse age range. Another limitation was the focus on specific variables such as PA and HV and UV, leaving aside other possible influencing factors, such as customer service or marketing campaigns. Likewise, through the statistical analyses applied to the hypothesized model, this study proved a lack of statistical significance in the relationship between PA and word-of-mouth.

It would be valuable to conduct longitudinal studies that examine how consumer perceptions change over time. For future research, it is suggested to balance the sample with respect to the status and the age range of participants, incorporating groups from various status and age brackets that better reflect the demographic diversity of fast-food consumers. This approach would enable more representative results and facilitate a greater generalization of the findings to the overall population. In addition, future research could explore the interaction of other variables, such as the impact of digital marketing and customer loyalty on word-of-mouth. Comparative studies between different cultures and restaurant types would also provide a broader understanding of these phenomena. In addition, future scholars are encouraged to develop hypothetical models that test the direct relationship of PA on WOM.

## Figures and Tables

**Table 1:** Demographics.

Characteristics	Category	N	%
Gender	Male	388	52.5%
	Female	351	47.5%
Marital Status	Single	349	47.2%
	Married	190	25.7%
	Separated	55	7.46%
	Widowed	101	13.67%
	Civil Union	44	5.97%
Age Range	18 to 25 years old	386	52%
	26 to 31 years old	128	17%
	32 to 37 years old	98	13%
	38 to 43 years old	88	12%
	44 to 50 years old	35	5%
	Over 50 years old	4	1%
Educational Level	Primary Education	11	1.48%
	Secondary Education	308	41.67%
	Technical Education	21	2.85%
	University Education	399	54%
Monthly Income	Less than 1 minimum wage	496	67.11%
	1 and 2 minimum wages	189	25.6%
	2 and 3 minimum wages	35	4.73%
	3 and 4 minimum wages	11	1.48%
	5 and 6 minimum wages	6	0.81%
	More than 6 minimum wages	2	0.27%
Weekly Spending on Fast Food	Up to CLP 30,000	145	19.64%
	Up to CLP 40,000	459	62.11%
	Up to CLP 50,000	88	11.9%
	Up to CLP 60,000	33	4.46%
	More than CLP 60,000	14	1.89%
N = 739 subjects (Average Age = 24 years)			

**Table 2:** Convergent validity and reliability.

Variable	Item	Loading Factor	Cronbach Alpha	Composed Reliability (CR)	Average Variance Extracted (AVE)
Physical Ambiance (PA)	PA1	0.769	0.875	0.885	0.728
	PA2	0.886			
	PA3	0.878			
	PA4	0.876			
Hedonic Value (HV)	HV1	0.805	0.889	0.899	0.753
	HV2	0.919			
	HV3	0.915			
	HV4	0.826			
Utilitarian Value (UV)	UV1	0.842	0.795	0.810	0.705
	UV2	0.857			
	UV3	0.821			
Consumer Satisfaction (CS)	CS1	0.932	0.936	0.936	0.887
	CS2	0.949			
	CS3	0.945			
Word-of-Mouth (WOM)	WOM1	0.944	0.943	0.944	0.898
	WOM2	0.959			
	WOM3	0.941			

**Table 3:** Discriminant validity.

	PA	HV	UV	CS	WOM
**PA**	0.854	0.813	0.540	0.726	0.653
**HV**	0.723	0.868	0.704	0.893	0.877
**UV**	0.461	0.608	0.840	0.637	0.670
**CS**	0.659	0.819	0.566	0.942	0.873
**WOM**	0.597	0.805	0.599	0.821	0.848

**Note:** Fornell and Larcker on the diagonal; HTMT values above the diagonal.

**Table 4:** Results of hypotheses testing.

Hypotheses	Relation	β	*p*-Values	Hypotheses
H1	PA-CS	0.136	***	Accepted
H1a	PA-HV	0.723	***	Accepted
H1b	PA-UV	0.461	***	Accepted
H2	HV-CS	0.656	***	Accepted
H2a	HV-WOM	0.348	***	Accepted
H3	UV-CS	0.105	***	Accepted
H3a	UV-WOM	0.123	***	Accepted
H4	CS-WOM	0.466	***	Accepted

R^2^ (HV) = 0.522; R^2^ (UV) = 0.211; R^2^ (SC) = 0.685; R^2^ (WOM) = 0.736; SRMR = 0.057 *** *p* < 0.001.

## Data Availability

The original data presented in the study are openly available at https://drive.google.com/drive/folders/13XoQ3uwo_QVjWQtEjRbUzB8vuCWczxMn?usp=sharing (accessed on 24 September 2024).

## Appendix A

**Table A1:** Question Survey.

Variable	Items
Physical Ambiance (PA) Adapted from [82] Jalilvand et al. (2017)	(AF1) The restaurant has visually attractive building exteriors and parking area.
	(AF2) The restaurant has a visually attractive dining area that is comfortable and easy to move around within.
	(AF3) The restaurant has appropriate music and illumination in keeping with its atmosphere.
	(AF4) The restaurant has clean and distinguished dining equipment (tables, chairs, dishes).
Hedonic Value (HV) [83] Adapted from Kisang et al. (2010)	(VH1) I have been coming to this restaurant ever since I had the desire.
	(VH2) Coming to this restaurant was fun and enjoyable.
	(VH3) The experience of eating at this restaurant was truly a pleasure.
	(VH4) During my experience at this restaurant, I felt excited as I looked through the menu for food.
Utilitarian Value (HV) [83] Adapted from Kisang et al. (2010)	(VU1) Eating out at this restaurant is convenient.
	(VU2) Eating out at this restaurant was pragmatic and economical.
	(VU3) It was not a waste of money to come and eat at this restaurant.
Consumer Satisfaction (CS) [82] Adapted from Jalilvand et al. (2017)	(SC1) I am satisfied with the service at this restaurant.
	(SC2) The restaurant always comes up to my expectations.
	(SC3) My experiences with the restaurant are excellent.
Word-of-Mouth (WOM) [82] Adapted from Jalilvand et al. (2017)	(WOM1) I say positive things about the restaurant to other people.
	(WOM2) I recommend the restaurant to someone who seeks your advice.
	(WOM3) I encourage friends and relatives to refer the restaurant.
